# Clinical and Haplotypic Variability of Slovenian *USH2A* Patients Homozygous for the c. 11864G>A Nonsense Mutation

**DOI:** 10.3390/genes10121015

**Published:** 2019-12-05

**Authors:** Andrej Zupan, Ana Fakin, Saba Battelino, Martina Jarc-Vidmar, Marko Hawlina, Crystel Bonnet, Christine Petit, Damjan Glavač

**Affiliations:** 1 Department of Molecular Genetics, Institute of Pathology, Faculty of Medicine, University of Ljubljana, 1000 Ljubljana, Slovenia; andrej.zupan@mf.uni-lj.si; 2 Eye Hospital, University Medical Centre Ljubljana, Grablovičeva 46, 1000 Ljubljana, Slovenia; ana.fakin@gmail.com (A.F.); martina.jarcvidmar@gmail.com (M.J.-V.); 3 Department of Otorhinolaryngology and Cervicofacial Surgery, University Medical Centre Ljubljana, 1000 Ljubljana, Slovenia; saba.battelino@kclj.si; 4 Faculty of Medicine, University of Ljubljana, 1000 Ljubljana, Slovenia; 5 Unité de Génétique et Physiologie de l’Audition, Institut Pasteur, 75015 Paris, France; crystel.bonnet@orange.fr (C.B.); cpetit@pasteur.fr (C.P.); 6 Unité Mixte de Recherche en Santé (UMRS) 1120, Institut National de la Santé et de la Recherche Médicale (INSERM), 75015 Paris, France; 7 Complexité du Vivant, Sorbonne Universités, Université Pierre et Marie Curie, Université Paris 06, 75005 Paris, France; 8 Institut de l’Audition, 75012 Paris, France; 9 Syndrome de Usher et Autres Atteintes Rétino-Cochléaires, Institut de la Vision, 75012 Paris, France; 10 Collège de France, 75005 Paris, France

**Keywords:** usher syndrome, founder effect, haplotype analysis, high resolution melting analysis

## Abstract

Purpose: to determine a detailed clinical and haplotypic variability of the Slovenian *USH2A* patients with homozygous c.11864G>A (p.Trp3955Ter) nonsense mutation and to develop sensitive, accurate and rapid screening test. Methods: Ten unrelated homozygous patients with detailed ophthalmological exam were included in our study. The High-Resolution Melting (HRM) method was developed for fast and reliable detection of the c.11864G>A mutation. Results: The c.11864G>A mutation represents the vast majority of pathogenic alleles in Slovenian USH2A-Usher syndrome population (84%). The median age of onset of nyctalopia was 16 years and all patients younger than 40 years had hyperautofluorescent rings on fundus autofluorescence imaging. The Kaplan Meier survival analysis showed a decline of central vision after the age of 40, with 50% patients reaching visual acuity (VA) ≤ 0.05 at the average age of 66 years visual field diameter less than 20° at the average age of 59 years. There was a relatively large phenotypic variability in the retinal and audiological phenotype. Analysis of the p.Trp3955Ter-homozygous patients revealed four different haplotypes, with the frequency of the most common haplotype ~65%. Disease severity did not correlate with the haplotype. Conclusions: According to the natural history of homozygous p.Trp3955Ter patients any therapy aimed to slow disease progression in these patients would be best started before the age of 40. Phenotypic variability suggests the presence of cis and/or trans factors outside the *USH2A* gene that are able to affect disease severity. High frequency of p.Trp3955Ter mutation in Slovenian *USH2A* gene pool appears to be initiated from different unrelated founders because of migrations from neighboring populations. The mutation on haplotype 2 seems to be the major founder allele.

## 1. Introduction

Usher syndrome (USH), an autosomal recessive genetic disease, is characterized by vision loss, hearing loss and balance problems [1,2,3]. Clinically the USH presents as Type I (USH1), Type II (USH2) and Type III (USH3) [4]. Apart from different genes causing USH1 (*MYO7A, USH1C, CDH23, PCDH15* and *USH1G*), USH2 (*USH2A, ADGRV1* and *WHRN*) and USH3 (*CLRN1*), the main clinical differences between the three types of Usher syndrome are the time of onset of retinitis pigmentosa (RP), the severity of hearing loss and the status of the vestibular function [1]. However, there is considerable variability within the subtypes resulting in overlapping phenotypes between USH1, USH2 and USH3 [1,4].

USH2 is associated with congenital moderate-to-severe hearing loss, normal vestibular function and onset of RP within the second decade of life [1,5,6]. Compound heterozygosity for mutations in *USH2A* is the most frequent cause of USH2 as well as isolated RP [7]. The *USH2A* gene was mapped to chromosome 1q41, spanning 800.05 kb with 72 exons and coding integral membrane protein usherin of approximately 5202 amino acids. It is believed that usherin is required for the long-term maintenance of retinal photoreceptors and for the development of cochlear hair cells [8].

Various genetic studies of USH2A patients revealed broad spectrum of different mutations in the *USH2A* gene [7,9,10,11,12,13,14,15,16,17,18,19]. Reports found that c.2299delG (p.Glu767Serfs*21) is the most common mutation in *USH2A*, accounting for the vast majority of pathologic alleles [12,20]. It was suggested that the widespread geographic distribution of the c.2299delG mutation is the result of an ancestral mutation that spread from South Europe throughout Europe and into the New World [18,21]. In the Slovenian *USH2A* population, however, c.2299delG is missing whereas c.11864G>A (p.Trp3955Ter) has been detected with a frequency of 84% [22]. The mutation c.11864G>A has been described in other European populations at low frequencies e.g., in France 2.5% and Italy 6.5%, with the exception of German population where it reaches 23% [23].

Although correlation between genotype and phenotype in Usher syndrome has been explored in different clinical studies [24,25,26,27,28], diagnosis and classification of different subtypes remain difficult for some patients, and the phenotypic variability across populations remains incompletely understood [3]. The phenotype–genotype correlation for the most frequent mutations in the *USH2A* gene can be of great importance in determining the prognosis for affected patients and can assist in genetic counselling, as well as guide the selection of patients for clinical trials, thus emphasizing the need for quick and reliable screening method for *USH2A* mutations.

The aim of this study was to determine clinical and haplotypic variability of the Slovenian homozygous p.Trp3955Ter -linked USH2A patients and to develop a sensitive, accurate and rapid screening test for the detection of the most common Slovenian *USH2A* mutation. Here, we utilize High-Resolution Melting (HRM), a quick and effective method in which the melting curve of an amplicon is analysed with fluorescent markers specific for double stranded DNA [29], to track the c. 11864G>A nonsense mutation in the Slovenian cohort.

## 2. Materials and Methods 

### 2.1. Patient Selection

The studied group consisted of unrelated homozygous p.Trp3955Ter patients (*N* = 10) who were identified from the Slovenian cohort of *USH2A* patients.

### 2.2. Ophthalmological Exam 

Ophthalmological exam included Snellen visual acuity (VA), color vision testing with Ishihara plates, Goldmann visual fields with II/4 stimulus (VF); fundus autofluorescence and optical coherence tomography (Spectralis, Heidelberg, Germany) and mesopic microperimetry (MP1, Nidek, Padova, Italy). Fundus autofluorescence patterns were categorized into ring, patch and atrophy, as previously described [30]. Kaplan Meier survival plots were performed for visual acuity and visual fields to determine the age when 50% patients reached legal blindness (VA ≤ 0.05 or VF diameter < 20°). Pure tone audiometry at 500, 1000, 2000 and 4000 Hz frequencies was performed and the hearing loss in dB averaged for each ear.

### 2.3. Sample Collection and DNA Extraction

Between 2010 and 2013, genetic testing for mutations was performed in 68 patients with Usher syndrome and their relatives when available, using a previously established method based on next generation sequencing [22]. Medical files of all the patients with Usher syndrome were reviewed for clinicopathological data. All patients, relatives or their trustees gave informed consent for genetic testing, in accordance with the regulation of our institute and National Medical Ethics Committee of the Republic of Slovenia. Genomic DNA was extracted from whole blood using a column-based purification kit (QIAamp DNA Blood Maxi kit, Qiagen, Hilden, Germany). DNA concentration was determined spectrophotometrically using NanoDrop-1000 (ThermoScientific, Wilmington, DE, USA).

In order to develop the HRM screening test, DNA samples tested positive for p.Trp3955Ter mutation and DNA samples tested negative for p.Trp3955Ter mutations were selected.

### 2.4. DNA Amplification and Genotyping

PCR amplification of exon 61 of the *USH2A* gene was performed with genomic DNA with one set of primers (Table 1). All PCRs were performed in a 15 μL reaction mixture containing 30 ng purified genomic DNA, 200 nM of each of the primers and 1× Type-It HRM PCR Kit (Qiagen, Hilden, Germany). PCR reactions were optimized: 5 minutes at 95 °C, followed by 45 cycles as follows: 10 seconds at 95 °C, 30 seconds at 56 °C, and 10 seconds at 72 °C, with detection of the fluorescence on the Green channel. Melting temperature was raised from 65 °C to 95 °C with a ramp of 0.02 °C per second and the detection of fluorescence on the HRM channel. Rotor-Gene Q 5plex HRM was used to perform the reactions (Qiagen, Hilden, Germany). Analysis of the HRM results was conducted using Rotor-Gene Q Series Software, 2.0.2 (Qiagen, Hilden, Germany).

For Sanger sequencing, the PCR products obtained with the HRM analysis were directly used for a sequencing reaction after being purified with Diffinity Rapid Tips (Sigma Aldrich, Taufkirchen, Germany). The ABI Prism 310 capillary sequencer (Applied Biosystems, Foster City, CA, USA) was used for sequencing. The sequencing reaction was performed using Big-Dye terminator chemistry version 1.1 (Applied Biosystems, Foster City, CA, USA). Electropherograms were analyzed with the Sequencing Analysis 5.2.0 software (Applied Biosystems, Foster City, CA, USA).

### 2.5. Haplotype Analysis

To establish c.11864G>A —linked haplotype, twelve single-nucleotide polymorphisms (SNPs) within *USH2A* gene, were selected (Table 2) as described previously [18,20,21]. Only homozygous p.Trp3955Ter patients (*N* = 10) were selected for the determination of the haplotype phase to avoid noise from other compound heterozygous mutations. The data were obtained from a previous study [22] and analysed as described above.

## 3. Results

**Figure 1:**
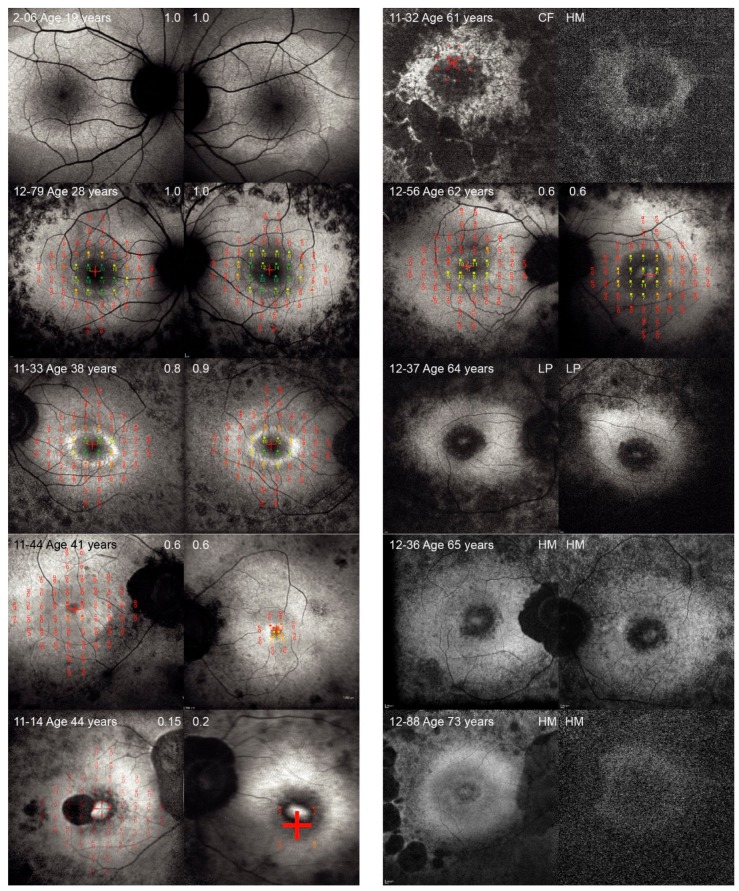
Fundus autofluorescence images of patients homozygous for p.Trp3955Ter mutation in *USH2A*, arranged by increasing age. Right and left eye are presented of each patient, Patient ID is stated in the bottom left corner and visual acuity is stated in the top corner for each eye. For those patients who had microperimetry performed, the results were overlayed on the fundus autofluorescence image using software image registration.

**Figure 2:**
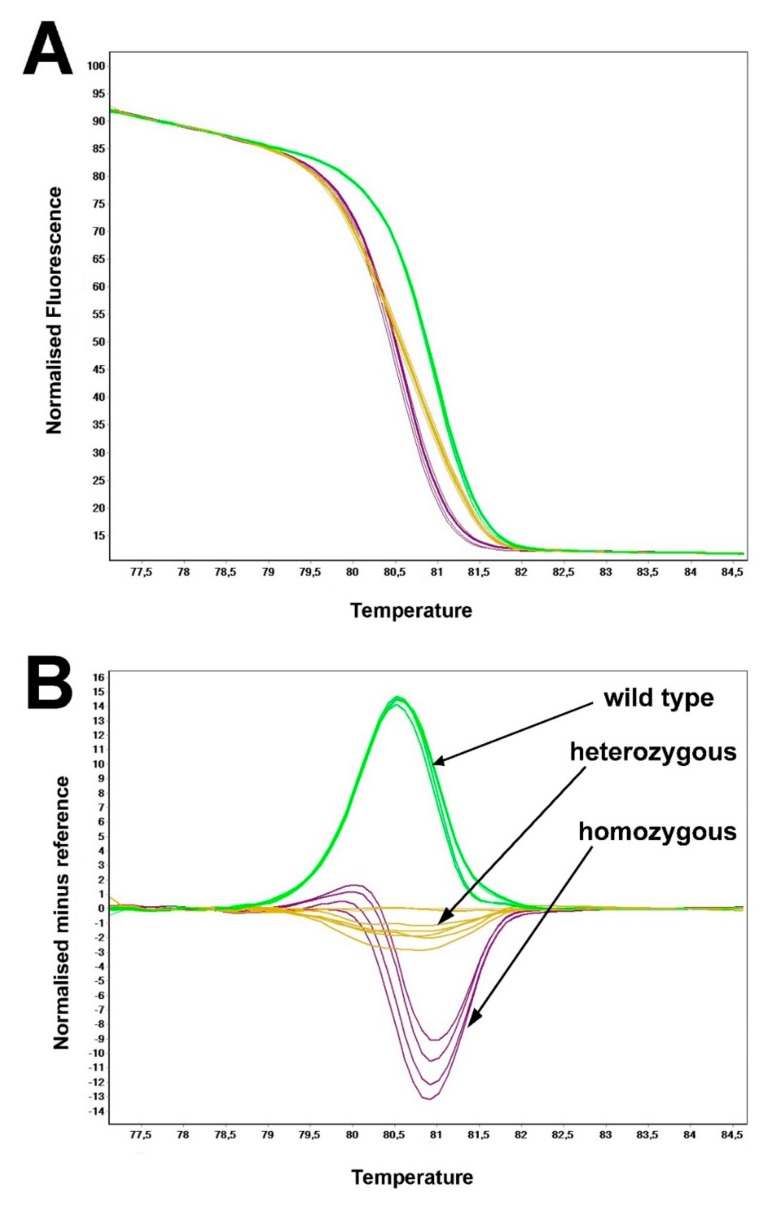
Normalized high-resolution melting profile (**A**) together with differential plot (**B**).

The phenotypic characteristics of the ten patients homozygous for c.11864G>A mutation are presented in Table 3 and Figure 1. The median age at the onset of nyctalopia was 16 years (range, 9–42 years). The median visual acuity of the better seeing eye at the time of examination (median age 41 years) was 0.6 (range, light perception to 1.0) and the median radius of the central visual field was 3° (range, 0°–15°). Kaplan Meier survival analysis predicted that 50% patients reached VA ≤ 0.05 at the average age of 66 years (95% CI, 61–71 years) and 50% patients reached visual field diameter less than 20° at the average age of 59 years (95% CI, 50–67 years) (Figure 2). Fundus autofluorescence imaging revealed bilateral hyperautofluorescent rings in five patients (median age 38, range 19–62 years) while five patients had either patch or atrophy patterns (median age 64, range 44–73 years). Cystoid macular oedema was noted in 4/10 (40%) patients. Microperimetry showed preserved retinal function inside the hyperautofluorescent rings. Pure tone audiometry at the median age of 33 years (range, 17–65 years), showed a median hearing loss of 58 dB (range, 56–98 dB) on the better hearing ear. One patient developed cholesteatoma and subsequently completely lost hearing on one ear.

To discriminate between different genotypes, HRM analysis was performed as follows: the melt plots were normalized and then transformed to difference plots, as a representation of the difference in fluorescence between samples to a selected control at each temperature transition. In order to produce a normalized melting graph, two normalized regions were selected; one encompassing the representative baseline data for the premelt phase and the other encompassing the representative data for the postmelt phase. The melting curve of exon 61 revealed a single melting peak with a temperature window spanning from 79.5 °C to 82 °C, suggesting a specific amplification. Normalized curve of the exon 61 (Figure 2A) revealed three distinct melting curves, each corresponding to three distinct genotypes (wild type, p.Trp3955Ter-heterozygous and p.Trp3955Ter-homozygous). The difference plot for the exon 61 was constructed using a genotype p.Trp3955Ter-heterozygous as a control sample (Figure 2B). The difference plot successfully differentiated between three different genotypes of codon 3955. Although all difference plot curves of exon 61 exhibited high degree of differentiation from each other, the highest difference could be observed between p.Trp3955Ter-homozygous mutation and a wild-type.

Haplotype analysis revealed four different haplotypes with the haplotype 2 being the most common at frequency of 65% and the haplotypes 3 and 4 being the least common at frequency of 5% (Table 2). Haplotype for each patient is shown in Figure 1. There was no obvious correlation between disease severity and haplotype. For example, patient 12–56 with the mildest disease shared the same haplotype (2/2) as two similarly aged patients with advanced disease.

## 4. Discussion

**Figure 3:**
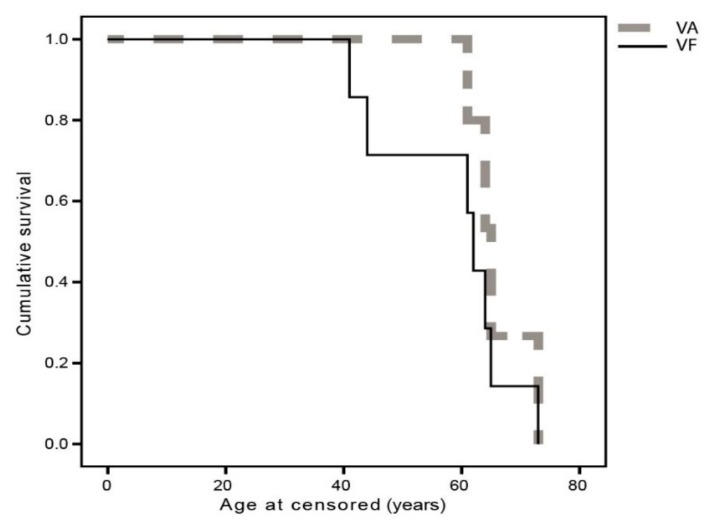
Kaplan Meier survival analysis plot for visual acuity (censored at VA ≤ 0.05) and II/4 Goldmann visual field diameter (censored at <20°).

While more than 600 distinct causing mutation in *USH2A* gene have been reported in relation to the Usher syndrome type 2 so far (https://databases.lovd.nl/shared/variants/USH2A/, the mutation p.Trp3955Ter in Slovenian population is of special interest due to its high frequency in this area. This paper delineates retinal and audiological phenotype of ten unrelated homozygotes for the same stop mutation p.Trp3955Ter in *USH2A*. The observed phenotype was consistent with the previous natural history reports of USH2 syndrome caused by *USH2A* mutations [30,31,32,33,34,35], i.e., presentation with nyctalopia in the second decade, narrowing of the visual field with age and progression from hyperfluorescent ring to patch and atrophy on fundus autofluorescent imaging Usher syndrome 1 and 2. Patients younger than 40 years had good central vision and hyperfluorescent rings reflecting preserved central retina whereas the decline in central visual function observed after the age of 40 (Figure 3—Kaplan Meier) suggests that, whenever possible, therapies aimed at slowing disease progression target young adult patients.

Considering that p.Trp3955Ter presumably undergoes nonsense mediated decay [36], none of these patients would have any functional USH2A protein. Nevertheless, there substantial phenotypic variability in the retinal and audiological phenotype suggests the presence of modulatory cis and/or trans factors outside of the *USH2A* gene. Nonsense mediated decay might be affected but it remains to be seen whether truncated proteins are functional. We also found that the disease severity varies across patients expressing the same mutation. Thus, one patient (case 12–56) showed a mild phenotype that was characterized by normal night vision before the age of 42 years, a preserved central retina at the age of 62 (as indicated by good visual acuity with 0.6 on the better eye) and hyperfluorescent ring pattern on fundus autoflurescence (Figure 1) whereas other patients exhibited disease progression to the fovea at this age. Further studies are needed to understand the variability of disease and caution needs to be taken when selecting patients for the clinical trials based on genotypes.

The development of fast and accurate method for detection of the most common Slovenian *USH2A* mutation p.Trp3955Ter enables pre-screening strategy for the fast identification of USH2A patients thus enabling faster turnaround time. The HRM method detects the mutation p.Trp3955Ter and differentiates between homozygous, heterozygous and wild type genotypes within less than three hours. Hence, it could be used to pre-screen for p.Trp3955Ter before proceeding to the more time-consuming and expensive Sanger or Next Generation Sequencing [37]. The use of PCR products of the HRM reaction for the sequencing reaction of positive samples can further reduce the turnaround time. However, because the sensitivity to the DNA concentration variability and quality can influence the melting profile of DNA sample, care must be taken within the preanalytical procedures, such as DNA isolation and quantification. Furthermore, PCR results must be inspected in real time in order to detect potential artefactual outliers that could influence the results via amplification and melt profile.

The c.11864G>A mutation represents the vast majority of pathogenic alleles in Slovenian USH2A-Usher syndrome population (84%), which is not the case in other populations [22]. In order to further investigate the causes for the high frequency of p.Trp3955Ter mutation in Slovenian USH2A population, p.Trp3955Ter-linked haplotypes were constructed. We found that Slovenian p.Trp3955Ter-homozygous patients exhibit four haplotypes, with the most common showing the frequency of 65%, revealing relatively homogenous haplotypic structure. Another observation was that the gene showed the greatest variability at the 5` end, unlike previous reports which suggested high recombinational activity at the 3` end [21]. The high frequency of p.Trp3955Ter mutation and its relatively homogenic haplotypic structure in non-related patients can be explained as a founder effect, presumably as a result of migrations from neighbouring populations. Interestingly, very recent study showed 50% of p.Trp3955Ter mutation in Russian cohort of USH2A patients, however their haplotype was not yet determined [38]. At least four different p.Trp3955Ter-linked haplotypes were observed in homozygous individuals, contributing the one common ancestral p.Trp3955Ter mutational event. The mutation on haplotype 2 seems to be the major founder allele. Additional inter-population studies across European populations will be helpful to delineate the genetic flow of *USH2A*. In advent of gene treatment, high uniformity of p.Trp3955Ter mutation implies possibility to treat larger group of patients, especially if the same mutation would show high prevalence in other regions of Central and Eastern Europe.

## 5. Conclusions

Our paper describes the clinical and haplotypic variability of the Slovenian homozygous p.Trp3955Ter-linked USH2A patients. Additionally we have developed a fast and reliable method for detection of the Slovenian most frequent *USH2A* mutation p.Trp3955Ter. The developed method can be implemented as a pre-screening method thus eliminating the need for more complex and expensive mutation detection methods and increasing the cost/effectiveness of the screening procedure for the Usher patients and family members. 

## Figures and Tables

**Table 1:** Oligonucleotide primer sequences for the HRM mutation analysis.

Exon	Primer Sequence		Final Concentration (nM)
**61**	Forward:	5’-TCTGGTCAGAAGGAGCCCTTGAAT-3’	200
	Reverse:	5’-AGGTGGAGCTTCCAGAGTTTGTGT-3’	

**Table 2:** Representation of p.Trp3955Ter associated haplotypes in homozygous USH2A patients.

	11–14	11–32	11–33	11–44	12–36	12–37	12–56	12–79	12–88	02–06
rs10779261	C	T	T	T	T	T	T	C/T	T	C/T
rs4253963	T	C	C	C	C/T	C	C	C/T	C	C/T
rs1805050	G	G	G	G	G	G	G	G	G	A/G
rs1324330	T	T	T	T	C/T	T	T	T	T	C/T
rs646094	A	A	A	A	A	A	A	A	A	A/C
rs1805049	T	T	T	T	C/T	T	T	T	T	C/T
rs6657250	G	G	G	G	G	G	G	G	G	G
rs10864219	A	A	A	A	A/G	A	A	A	A	A
rs10864198	T	T	T	T	T	T	T	T	T	T
rs11120616	G	G	G	G	G	G	G	G	G	G
rs35309576	T	T	T	T	T	T	T	T	T	T
rs2820718	C	C	C	C	C	C	C	C	C	C
Haplotype	Ht1	Ht2	Ht2	Ht2	Ht2/3	Ht2	Ht2	Ht1/2	Ht2	Ht1/4

**Table 3:** Phenotypic characteristics of homozygous patients for p.Trp3955Ter.

Patient	Sex	Age at Onset of Nyctalopia (Years)	Age at Eye Exam (Years)	VA (RE, LE)	Ishihara (RE, LE; N Out of 15 Plates Seen)	Goldmann II4 Radius (RE, LE) (Degrees)	Fundus Autofluorescence Pattern (RE, LE)	Age at Audiogram (Years)	Average Hearing Loss Accross (R,L) (dB)
2–06	Male	12	19	1.0. 1.0	N/A	N/A	Ring, Ring	21	56, 60
12–79	Male	22	28	1.0, 1.0	14, 14	10, 15	Ring, Ring	17	66, 68
11–33	Male	9	38	0.8, 0.9	13, 13	11, 10	Ring, Ring	30	130*, 80
11–44	Male	13	41	0.6, 0.6	1, 1	3, 3	Ring, Ring	42	71, 65
11–14	Female	12	44	0.15, 0.2	0, 0	0, 0	Patch, Patch	44	59, 68
11–32	Male	10	61	CF, HM	0, 0	2, 1	Atrophy, Atrophy	57	75, 75
12–56	Male	42	62	0.6, 0.2	3, 1	6, 6	Ring, Ring	N/A	N/A
12–37	Male	16	64	LP, LP	0, 0	0, 0	Patch, Patch	65	59, 61
12–36	Female	35	65	HM, HM	0, 0	0, 0	Patch, Patch	N/A	N/A
12–-8	Female	4	73	HM, HM	0, 0	N/A	Patch, Atrophy	63	89, 98

RE = Right eye, LE = Left eye, CF = Counting fingers, HM = Hand motion, LP = Light perception, N/A = Not applicable; * Cholesteatoma. Patients are arranged by age at ophthalmological exam.
